# Risk for *Clostridiodes difficile* Infection among Older Adults with Cancer

**DOI:** 10.3201/eid2509.181142

**Published:** 2019-09

**Authors:** Mini Kamboj, Renee L. Gennarelli, Jennifer Brite, Kent Sepkowitz, Allison Lipitz-Snyderman

**Affiliations:** Memorial Sloan Kettering Cancer Center, New York, New York, USA (M. Kamboj, R.L. Gennarelli, J. Brite, K. Sepkowitz, A. Lipitz-Snyderman);; Weill Cornell Medical College, New York (M. Kamboj, K. Sepkowitz)

**Keywords:** Clostridium difficile, Clostridiodes difficile, cancer, age, population, epidemiology, bacteria, United States, enteric infections

## Abstract

Incidence was higher for patients with cancer, independent of prior healthcare-associated exposure.

## Medscape CME ACTIVITY

In support of improving patient care, this activity has been planned and implemented by Medscape, LLC and Emerging Infectious Diseases. Medscape, LLC is jointly accredited by the Accreditation Council for Continuing Medical Education (ACCME), the Accreditation Council for Pharmacy Education (ACPE), and the American Nurses Credentialing Center (ANCC), to provide continuing education for the healthcare team.

Medscape, LLC designates this Journal-based CME activity for a maximum of 1.00 ***AMA PRA Category 1 Credit(s)*™**. Physicians should claim only the credit commensurate with the extent of their participation in the activity.

Successful completion of this CME activity, which includes participation in the evaluation component, enables the participant to earn up to 1.0 MOC points in the American Board of Internal Medicine's (ABIM) Maintenance of Certification (MOC) program. Participants will earn MOC points equivalent to the amount of CME credits claimed for the activity. It is the CME activity provider's responsibility to submit participant completion information to ACCME for the purpose of granting ABIM MOC credit.

All other clinicians completing this activity will be issued a certificate of participation. To participate in this journal CME activity: (1) review the learning objectives and author disclosures; (2) study the education content; (3) take the post-test with a 75% minimum passing score and complete the evaluation at http://www.medscape.org/journal/eid; and (4) view/print certificate.

### Release date: August 21, 2019; Expiration date: August 21, 2020

### Learning Objectives

Upon completion of this activity, participants will be able to:

- Describe findings of factors affecting risk for *Clostridiodes difficile* infection (CDI) in older adults, according to a retrospective cohort study analysis using population-based SEER-Medicare data for 2011
- Determine findings of factors affecting risk for CDI in older adults, according to a nested case-control study analysis using population-based SEER-Medicare data for 2011
- Identify clinical implications of risk factors for CDI in older adults, according to a retrospective cohort study with a nested case-control analysis using population-based SEER-Medicare data for 2011

### CME Editor

**P. Lynne Stockton Taylor, VMD, MS, ELS(D), **Technical Writer/Editor, Emerging Infectious Diseases. *Disclosure: P. Lynne Stockton Taylor, VMD, MS, ELS(D), has disclosed no relevant financial relationships.*

### CME Author

**Laurie Barclay, MD,** freelance writer and reviewer, Medscape, LLC. *Disclosure: Laurie Barclay, MD, has disclosed no relevant financial relationships.*

### Authors

*Disclosure:* ***Mini Kamboj, MD; Renee Lynn Gennarelli, MS; Jennifer Brite, DrPH; Kent Sepkowitz, MD;*** *and* ***Allison Lipitz-Snyderman, PhD,*** *have disclosed no relevant financial relationships.*
