# Correction: Vol. 25, No. 9

**DOI:** 10.3201/eid2510.C22510

**Published:** 2019-10

**Keywords:** errata, erratum, correction

*Clostridioides* was misspelled in Risk for *Clostridioides difficile* Infection among Older Adults with Cancer (M. Kamboj et al.). The article has been corrected online (https://wwwnc.cdc.gov/eid/article/25/9/18-1142_article).

