# Supplementary material for: Risk for Clostridiodes difficile Infection among Older Adults with Cancer
Source: Emerg Infect Dis. 2019 Sep;25(9):1683–9. doi: 10.3201/eid2509.181142 (PMC6711221; doi:10.3201/eid2509.181142)
Supplement: Related texts

# Research article

## Abstract

To assess whether risk for *Clostridiodes difficile* infection (CDI) is higher among older adults with cancer, we conducted a retrospective cohort study with a nested case–control analysis using population-based Surveillance, Epidemiology, and End Results–Medicare linked data for 2011. Among 93,566 Medicare beneficiaries, incident CDI and odds for acquiring CDI were higher among patients with than without cancer. Specifically, risk was significantly higher for those who had liquid tumors and higher for those who had recently diagnosed solid tumors and distant metastasis. These findings were independent of prior healthcare-associated exposure. This population-based assessment can be used to identify targets for prevention of CDI.

Healthcare-associated infections are common and often preventable infections that lead to high morbidity and mortality rates (*1*). The bacterium *Clostridiodes difficile* (formerly *Clostridium difficile*) causes inflammation of the colon, which commonly causes diarrhea. In the United States, *C. difficile* infection (CDI) is the leading cause of healthcare-associated infections (*2*).

Among the general population, the risk for CDI among older adults (>65 years of age) is up to 26 times greater than that among younger adults (*2*). The effects of this infection are also worse for older persons. In 2015, CDI ranked as the 18th leading cause of death for persons >65 years of age (*3*). Although persons in this age group accounted for only 48% of CDI cases, they accounted for ≈87% of deaths from CDI (*3*,*4*).

For older adults, the risk for CDI may be amplified even further by cancer or its treatment. Common factors that influence risk for CDI in general are advanced age, diminished humoral immune response, healthcare-associated exposures, and extended receipt of antimicrobial drugs, all of which are widely prevalent aspects of oncologic and supportive care (*5*). In addition, exposure to certain cytotoxic chemotherapeutic drugs has been postulated to induce a loss of resistance to colonization with *C. difficile*, although the mechanisms are unclear (*6*,*7*). Yet, the complex relationship between cancer in older adults and CDI remains weakly characterized at the population level (*8*,*9*).

Our objective with this study was to determine whether the risk for CDI is higher among older adults with cancer than among older adults without cancer. A population-level assessment of cancer-specific incidence of CDI among older adults would not only increase our knowledge of the disease epidemiology in this specialized population but could also guide primary prevention strategies, focusing on those at greatest risk for development of this complication.

## Methods

### Study Design

This study was considered exempt research by the institutional review board of Memorial Sloan Kettering Cancer Center (New York, New York, USA). Use of Surveillance, Epidemiology, and End Results (SEER)–Medicare data for this study was approved by the National Cancer Institute (Bethesda, MD, USA).

We conducted a retrospective cohort study with a nested case–control analysis and used population-based SEER-Medicare–linked data to assess CDI occurrence in 2011. The National Cancer Institute–sponsored SEER program includes regions that cover ≈28% of the US population. SEER registries include information about cancer site, initial treatments, and active follow-up for death. The SEER-Medicare dataset links cancer registry files with Medicare enrollment information and claims for Medicare beneficiaries with a diagnosis of cancer. Compared with the US older adult population, the SEER-Medicare cohort has a similar age and sex distribution, slightly higher proportion of patients residing in urban and high-income areas, and smaller proportion of nonwhite persons (*10*). We used the random 5% sample of Medicare beneficiaries who reside in SEER regions to identify non–cancer patients.

### Study Participants

We included Medicare beneficiaries with and without a cancer diagnosis. For beneficiaries with cancer, we included those with solid (breast, colon, lung, prostate, and head and neck cancers) and liquid (lymphoma, myeloma, leukemia) tumors diagnosed during 2006–2010. We required that participants had been >66 years of age at the time of diagnosis to allow for 1 year of claims to assess prior healthcare use and CDI diagnoses. For inclusion in the non–cancer patient sample, we required that Medicare beneficiaries had been >66 years of age at the start of 2011 with no history of cancer.

All patients included in the cohort had to have been hospitalized >1 time in 2011. We excluded those for whom Medicare Parts A and B coverage during 2010–2011 was incomplete and those who had a diagnosis of CDI in 2010 (the year before our study year).

### Variables

CDI diagnoses were based on code 008.45 of the International Classification of Diseases, 9th Revision, on an inpatient claim. Because of a lack of dates for diagnosis codes on inpatient claims, the admission date of the first claim with a CDI diagnosis was used as the CDI date. We examined the following patient characteristics: age, sex, race, geographic region, urban/rural location, hospitalizations from the prior year (within 12 months before the index date), and stays in a skilled nursing facility from the prior year (within 12 months before the index date). Cancer status was categorized as cancer versus no cancer, as solid versus liquid tumors, and by individual disease types. For cancer patients, stage at diagnosis was categorized according to the SEER historic staging variable: in situ, local/regional, or distant or unknown.

### Cohort Analysis

We determined CDI incidence in 2011 for hospitalized patients with and without cancer. Incidence was reported as the percentage of the cohort in whom CDI developed during the study period. We also examined the overall proportion of patients with CDI and CDI incidence by patient characteristics.

### Nested Case–Control Analysis

We included all patients with a diagnosis of CDI in 2011 in the nested case–control analysis; the index date was defined as the date of first CDI diagnosis in 2011. We randomly selected 5 control participants for each case-patient, matched by age (±1 year) at study start date (January 1, 2011) and sex. The index date for each control was the date of CDI for the matched case-patient. Each matched control must have had follow-up through at least the case-patient’s index date with no occurrence of CDI from study start date through index date. Case-patients could not be their own controls, but a case-patient was eligible to be a control for another case-patient if the criteria were met.

We compared the cancer status and other characteristics of the case-patients and controls. We used conditional logistic regression models for individually matched case-patients and controls to obtain the odds of CDI among those with a cancer diagnosis. We calculated adjusted and unadjusted odds ratios (ORs) for CDI by tumor type, stage at diagnosis, and year of diagnosis; the reference group was non–cancer patients. The stage-specific model was run for solid tumors with the following 3 SEER categories: in situ, locoregional, or distant versus no cancer (reference group). Odds of CDI for patients with liquid tumors were not reported because liquid tumors are staged as distant disease. Odds of CDI occurrence were also calculated by prior hospitalizations and prior stays at a skilled nursing facility. All conditional logistic regression models included as covariates patient race, geographic region, urban/rural location, cancer status, prior hospitalizations, and prior stays at a skilled nursing facility. For the case–control analysis, we used stays within 12 months before a patient’s index date to define prior hospitalizations and prior skilled nursing facility stays.

To examine associations among cancer patients who were likely to actively receive treatment, we also repeated the analysis for patients whose cancer was diagnosed during 2009–2010 only and those without a cancer diagnosis. We used the same method to obtain new matched case-patients and controls for this analysis. We considered p<0.05 to be statistically significant. All analyses were conducted by using SAS version 9.4 (SAS Institute Inc., https://www.sas.com).

## Results

### Cohort Analysis

For the 93,566 beneficiaries in the study cohort, 2.6% had CDI during the study period. In unadjusted analyses, a higher proportion of patients with cancer had CDI (2.8%) than did patients without cancer (2.4%). The proportion with CDI was higher among female patients and patients living in the Northeast and metropolitan areas. When we analyzed by 5-year age intervals, we found an incremental increase in CDI risk, from 1.9% among patients 66–69 years of age to 2.9% among patients >85 years of age (Table 1).

**Table 1:** *Clostridiodes difficile* infection, by cohort characteristics, in study of risk among older adults with cancer, United States

Characteristic	Total no.	% Patients with *C. difficile* infection
Total	93,566	2.6
Cancer status		
Cancer	47,323	2.8
No cancer	46,243	2.4

Age, y		
66–69	12,001	1.9
70–74	20,691	2.4
75–79	20,155	2.6
80–84	18,651	2.8
>85	22,068	2.9

Sex		
M	42,330	2.4
F	51,236	2.8

Race		
White	77,831	2.6
Black	8,885	2.6
Other/unknown	6,850	2.2

Urban/rural		
Metropolitan	76,808	2.8
Not metropolitan	16,703	1.8

US region		
West	36,726	2.5
Midwest	12,086	3.0
South	24,980	2.0
Northeast	19,774	3.3

### Nested Case–Control Analysis

For the 2,421 case-patients with CDI, we identified 12,105 matched controls. A higher proportion of case-patients (54%) than controls (49%) had cancer. The distribution of race was similar among case-patients and controls, but distribution by geography differed slightly. Higher proportions of case-patients than controls had been hospitalized multiple times before or had stayed in a skilled nursing facility (Table 2).

**Table 2:** Characteristics of case-patients and controls in study of *Clostridiodes difficile* infection in older adults with cancer, United States

Characteristic	% Total case-patients, n = 2,421	% Total controls, n = 12,105
Cancer diagnosis		
No cancer	46	51
Solid tumor	43	43
Liquid tumor	12	7

Age, y*		
66–69	10	10
70–74	20	21
75–79	21	21
80–84	22	22
>85	27	27

Sex*		
M	41	41
F	59	59

Race		
White	84	83
Black	10	9
Other/unknown	6	7

Urban/rural		
Metropolitan	87	83
Not metropolitan	13	17

US region		
West	38	40
Midwest	15	13
South	20	26
Northeast	27	21

Prior hospitalizations†		
0	20	37
1	18	32
2	19	16
3	12	6
4	10	4
>5	22	5

Prior skilled nursing facility stay†		
No	56	83
Yes	44	18

*Case-patients with *Clostridioides difficile* infection were matched to controls without *C. difficile* infection, by age and sex. †Stays within 12 mo before a patient’s index date were used to define prior hospitalizations and prior skilled nursing facility stays.

The odds of CDI developing were higher among cancer patients than non–cancer patients (adjusted OR 1.15, 95% CI 1.04–1.26; p = 0.005). When cancer was subdivided into solid and liquid tumor types, having a solid tumor was not significantly associated with an increased risk for CDI compared with having no cancer diagnosis (adjusted OR 1.05, 95% CI 0.95–1.16); an underlying diagnosis of a liquid tumor was significantly associated with increased risk for CDI compared with no cancer diagnosis (adjusted OR 1.74, 95% CI 1.48–2.06; p<0.001). When we restricted the analysis to only patients who had received a cancer diagnosis within the past 2 years or no cancer diagnosis at all, we found that both solid and liquid tumor types were significantly associated with increased odds of CDI compared with no cancer diagnosis (Tables 3, 4). Regardless of a cancer diagnosis, >2 prior hospitalizations or a prior stay at a skilled nursing facility were each associated with increased odds of CDI occurrence (Table 3).

**Table 3:** Odds ratios of infection occurrence, nested case–control analysis in study of *Clostridiodes difficile* infection in older adults with cancer

Characteristic	Primary analysis: cancer patients and non–cancer patients*			
	Unadjusted OR (95% CI)	p value	Adjusted OR (95% CI)†	p value
Tumor type				
None	1.00		1.00	
Solid	1.13 (1.03–1.24)	0.01	1.05 (0.95–1.16)	0.32
Liquid	1.94 (1.67–2.26)	<0.001	1.74 (1.48–2.06)	<0.001

Prior hospitalizations				
0	1.00		1.00	
1	1.11 (0.96–1.28)	0.15	1.07 (0.93–1.23)	0.37
2	2.33 (2.02–2.69)	<0.001	1.83 (1.56–2.14)	<0.001
3	3.55 (2.99–4.20)	<0.001	2.68 (2.22–3.23)	<0.001
4	4.93 (4.09–5.96)	<0.001	3.49 (2.81–4.31)	<0.001
>5	8.63 (7.39–10.09)	<0.001	5.76 (4.74–6.99)	<0.001

Prior skilled nursing facility stay				
No	1.00		1.00	
Yes	3.93 (3.56–4.33)	<0.001	1.59 (1.39–1.82)	<0.001

*For cancer patients, diagnosis was made during 2006–2010. OR, odds ratio. †Adjusted odds of *C. difficile* infection based on conditional logistic regression model. Adjusted for age, sex, race, geographic region, urban/rural location, prior hospitalizations, and prior skilled nursing facility stays. Separate models were conducted for all patients with a cancer diagnosed 2006–2010 and noncancer patients, and for recently diagnosed cancer patients (diagnosis 2009–2010) and non–cancer patients.

**Table 4:** Adjusted odds ratios of *Clostridiodes difficile* infection occurrence, nested case–control analysis

Characteristic	Subanalysis: recently diagnosed cancer patients and non–cancer patients*			
	Unadjusted OR (95% CI)	p value	Adjusted OR (95% CI)†	p value
Tumor type				
None	1.00		1.00	
Solid	1.36 (1.22–1.53)	0.01	1.24 (1.10–1.40)	<0.001
Liquid	2.22 (1.83–2.69)	<0.001	1.84 (1.49–2.26)	<0.001

Prior hospitalizations				
0	1.00		1.00	
1	1.13 (0.96–1.33)	0.13	1.07 (0.91–1.26)	0.40
2	2.28 (1.94–2.68)	<0.001	1.73 (1.44–2.08)	<0.001
3	3.17 (2.60–3.86)	<0.001	2.32 (1.86–2.88)	<0.001
4	4.31 (3.48–5.33)	<0.001	2.92 (2.29–3.72)	<0.001
>5	8.34 (7.00–9.94)	<0.001	5.25 (4.21–6.55)	<0.001

Prior skilled nursing facility stay				
No	1.00		1.00	
Yes	3.80 (3.40–4.24)	<0.001	1.64 (1.41–1.92)	<0.001

*For cancer patients, diagnosis was made during 2009–2010. OR, odds ratio. †Adjusted odds of CDI based on conditional logistic regression model. Adjusted for age, sex, race, geographic region, urban/rural location, prior hospitalizations, and prior skilled nursing facility stays. Separate models were conducted with all patients with a cancer diagnosis during 2006–2010 and noncancer patients, and for recently diagnosed cancer patients (those diagnosed 2009–2010) and noncancer patients.

Findings from our adjusted stage-specific model demonstrated that the odds of CDI were higher for patients with a solid tumor than for those with no cancer (Figure). Odds of CDI were also higher for patients with solid cancer at an unknown stage at diagnosis, although this finding probably depends on the constructs of the historic staging variable in SEER. Odds of CDI did not differ significantly for cancer patients with in situ or local/regionalized solid tumors at diagnosis, compared with non–cancer patients. 

**Figure:** Adjusted (A) and unadjusted (B) odds of CDI for cancer patients with solid tumor compared with non–cancer patients. Stage is based on Surveillance, Epidemiology, and End Results historic staging variable. aOR estimates were generated from a logistic regression model adjusted for age, sex, race geographic region, urban/rural location, prior hospitalization, and prior skilled nursing stay. Non–cancer patients serve as the reference group, indicated by the reference line at 1.0. Error bars indicate 95% CIs. aOR, adjusted odds ratio; CDI, *Clostridioides difficile* infection; OR, odds ratio.

## Discussion

The main findings of our study, derived from a population-based cohort of Medicare beneficiaries >65 years of age, indicate that the risk for CDI is greater for older adults undergoing treatment for cancer than for age-matched controls. We show that much of this excess risk is associated with the type of underlying cancer and advanced disease and remains independent of prior healthcare-associated exposure from inpatient and skilled nursing facility stays. Risk was highest for patients with hematologic malignancies. In comparison, for solid tumor patients, odds of developing CDI were higher only for those with a recent cancer diagnosis and those with distant metastasis at diagnosis.

Our findings collectively expand knowledge of how cancer diagnosis affects CDI-associated illness among older adults. Reports of CDI in the absence of exposure to antimicrobial drugs has been well described for patients receiving cytotoxic chemotherapy (*11*), plausibly related to intestinal dysbiosis caused by these agents. In our study, the occurrence of CDI seemed to be influenced by active oncologic therapy among patients with solid tumors, as suggested by the heightened risk at the time of new diagnosis, when the intensity of treatment is expected to be greater. Similarly, the CDI risk for patients with solid tumors was also higher for those with distant site metastases at the time of diagnosis, underscoring the complexities in management of advanced cancers. We cannot determine from this study whether these associations are driven by type of cancer treatment, excessive use of antimicrobial drugs, or a consequence of immunologic and microbial perturbation associated with cancer treatment in persons of advanced age. We were unable to quantify antecedent exposure to antimicrobial drugs, a major driver of CDI (*12*–*15*); the coexistence of several confounding factors makes this distinction particularly challenging. When we made risk adjustments for race, geographic region, type of healthcare facility, and number of prior healthcare facility stays, the odds of CDI in patients with liquid tumors were significantly higher. Similar to previous studies of non–cancer populations, we found an incremental increase in CDI risk with number of hospitalizations; risk for those with >3 hospital stays was 3-fold higher (*12*,*16*).

Symptoms of primary and recurrent CDI can be particularly debilitating in persons with advanced age and are frequently associated with postinfectious irritable bowel syndrome (*5*). In patients receiving concomitant cancer therapy, CDI symptoms are often indistinguishable from the gastrointestinal side effects of chemotherapy, radiation, and newer immunotherapies (*17*). For these complex reasons, CDI during cancer treatment can lead to delays in future chemotherapy or radiation cycles and have been shown to negatively affect eligibility for curative treatment options (*18*). In addition, the estimated surplus healthcare-associated costs of primary CDI in patients with advanced age is estimated to be around US $37,000 and for patients with immunocompromising conditions US $16,000, almost 2-fold higher than costs of CDI for patients without immunocompromising conditions (*19*). The wide-ranging effects of CDI in this population warrants assessment of primary prevention strategies (*20*–*22*). Our study defines the subset of older adults with cancer who would probably benefit the most from such therapies to minimize the vulnerability to CDI during cancer treatment.

The strength of our study is that it provides population-based quantitative estimates of the differential effects of CDI among older adults with and without cancer. Despite the many advantages of the SEER-Medicare dataset, our study has limitations: we were able to measure CDI-related disease burden only in the hospital setting, and community-acquired (or community-onset) cases were not included unless they resulted in a hospitalization. Although we excluded patients who received a CDI diagnosis in 2010, case-patients do not necessarily represent those with primary infections. We used only the first Medicare claim with a CDI diagnosis; outpatient diagnoses would be missed and recurrent disease could potentially be represented as incident CDI. In addition, we did not measure relapsing CDI.

In summary, the burden of CDI among older adults is greater among those with underlying cancer. Regardless of prior healthcare-associated exposure, risk for CDI was highest for patients with hematologic malignancy and those with recent diagnosis of solid tumor or distant metastatic disease at diagnosis. These findings can be used to guide CDI prevention strategies.

## References

[1] Magill SS, Edwards JR, Bamberg W, Beldavs ZG, Dumyati G, Kainer MA, et al.; Emerging Infections Program Healthcare-Associated Infections and Antimicrobial Use Prevalence Survey Team. Multistate point-prevalence survey of health care-associated infections. N Engl J Med. 2014;370:1198–208. 10.1056/NEJMoa130680124670166PMC4648343

[2] Lessa FC, Mu Y, Bamberg WM, Beldavs ZG, Dumyati GK, Dunn JR, et al. Burden of *Clostridium difficile* infection in the United States. N Engl J Med. 2015;372:825–34. 10.1056/NEJMoa140891325714160PMC10966662

[3] Murphy SL, Xu J, Kochanek KD, Curtin SC, Arias E. Deaths: Final Data for 2015. Natl Vital Stat Rep. 2017;66:1–75.29235985

[4] Centers for Disease Control and Prevention. 2015 Annual Report for the Emerging Infections Program for *Clostridium difficile* Infection [cited 2018 Jun 18]. https://www.cdc.gov/hai/eip/Annual-CDI-Report-2015.html

[5] Neemann K, Freifeld A. *lostridium difficile*–associated diarrhea in the oncology patient. J Oncol Pract. 2017;13:25–30. 10.1200/JOP.2016.01861428084880

[6] Anand A, Glatt AE. *Clostridium difficile* infection associated with antineoplastic chemotherapy: a review. Clin Infect Dis. 1993;17:109–13. 10.1093/clinids/17.1.1098353229

[7] Loo VG, Bourgault AM, Poirier L, Lamothe F, Michaud S, Turgeon N, et al. Host and pathogen factors for *Clostridium difficile* infection and colonization. N Engl J Med. 2011;365:1693–703. 10.1056/NEJMoa101241322047560

[8] Chopra T, Alangaden GJ, Chandrasekar P. *Clostridium difficile* infection in cancer patients and hematopoietic stem cell transplant recipients. Expert Rev Anti Infect Ther. 2010;8:1113–9. 10.1586/eri.10.9520954878

[9] Kamboj M, Son C, Cantu S, Chemaly RF, Dickman J, Dubberke E, et al. Hospital-onset *Clostridium difficile* infection rates in persons with cancer or hematopoietic stem cell transplant: a C3IC network report. Infect Control Hosp Epidemiol. 2012;33:1162–5. 10.1086/66802323041818PMC3670420

[10] Warren JL, Klabunde CN, Schrag D, Bach PB, Riley GF. Overview of the SEER-Medicare data: content, research applications, and generalizability to the United States elderly population. Med Care. 2002;40(Suppl):IV-3–18. 10.1097/00005650-200208001-0000212187163

[11] Kamthan AG, Bruckner HW, Hirschman SZ, Agus SG. *Clostridium difficile* diarrhea induced by cancer chemotherapy. Arch Intern Med. 1992;152:1715–7. 10.1001/archinte.1992.004002001390251497405

[12] Cohen SH, Gerding DN, Johnson S, Kelly CP, Loo VG, McDonald LC, et al.; Society for Healthcare Epidemiology of America; Infectious Diseases Society of America. Clinical practice guidelines for Clostridium difficile infection in adults: 2010 update by the society for healthcare epidemiology of America (SHEA) and the infectious diseases society of America (IDSA). Infect Control Hosp Epidemiol. 2010;31:431–55. 10.1086/65170620307191

[13] McFarland LV, Clarridge JE, Beneda HW, Raugi GJ. Fluoroquinolone use and risk factors for *Clostridium difficile*-associated disease within a Veterans Administration health care system. Clin Infect Dis. 2007;45:1141–51. 10.1086/52218717918075

[14] Nelson DE, Auerbach SB, Baltch AL, Desjardin E, Beck-Sague C, Rheal C, et al. Epidemic *Clostridium difficile*-associated diarrhea: role of second- and third-generation cephalosporins. Infect Control Hosp Epidemiol. 1994;15:88–94. 10.2307/301455378201240

[15] Stevens V, Dumyati G, Fine LS, Fisher SG, van Wijngaarden E. Cumulative antibiotic exposures over time and the risk of *Clostridium difficile* infection. Clin Infect Dis. 2011;53:42–8. 10.1093/cid/cir30121653301

[16] Tabak YP, Johannes RS, Sun X, Nunez CM, McDonald LC. Predicting the risk for hospital-onset *Clostridium difficile* infection (HO-CDI) at the time of inpatient admission: HO-CDI risk score. Infect Control Hosp Epidemiol. 2015;36:695–701. 10.1017/ice.2015.3725753106PMC5768429

[17] Kamboj M, Brite J, Aslam A, Kennington J, Babady NE, Calfee D, et al. Artificial differences in *Clostridium difficile* infection rates associated with disparity in testing. Emerg Infect Dis. 2018;24:584–7. 10.3201/eid2403.17096129460760PMC5823336

[18] Hautmann MG, Hipp M, Kölbl O. *Clostridium difficile*-associated diarrhea in radiooncology: an underestimated problem for the feasibility of the radiooncological treatment? Radiat Oncol. 2011;6:89. 10.1186/1748-717X-6-8921806799PMC3160362

[19] Zhang D, Prabhu VS, Marcella SW. Attributable healthcare resource utilization and costs for patients with primary and recurrent *Clostridium difficile* infection in the United States. Clin Infect Dis. 2018;66:1326–32. 10.1093/cid/cix102129360950PMC5905590

[20] Wilcox MH, Gerding DN, Poxton IR, Kelly C, Nathan R, Birch T, et al.; MODIFY I and MODIFY II Investigators. MODIFY I and MODIFY II Investigators. Bezlotoxumab for prevention of recurrent *Clostridium difficile* infection. N Engl J Med. 2017;376:305–17. 10.1056/NEJMoa160261528121498

[21] Lewis BB, Pamer EG. Microbiota-based therapies for *Clostridium difficile* and antibiotic-resistant enteric infections. Annu Rev Microbiol. 2017;71:157–78. 10.1146/annurev-micro-090816-09354928617651

[22] Martin J, Wilcox M. New and emerging therapies for *Clostridium difficile* infection. Curr Opin Infect Dis. 2016;29:546–54. 10.1097/QCO.000000000000032027753689

# Research article

## Earning CME Credit

To obtain credit, you should first read the journal article. After reading the article, you should be able to answer the following, related, multiple-choice questions. To complete the questions (with a minimum 75% passing score) and earn continuing medical education (CME) credit, please go to http://www.medscape.org/journal/eid. Credit cannot be obtained for tests completed on paper, although you may use the worksheet below to keep a record of your answers. 

You must be a registered user on http://www.medscape.org. If you are not registered on http://www.medscape.org, please click on the “Register” link on the right hand side of the website. 

Only one answer is correct for each question. Once you successfully answer all post-test questions, you will be able to view and/or print your certificate. For questions regarding this activity, contact the accredited provider, CME@medscape.net. For technical assistance, contact CME@medscape.net. American Medical Association’s Physician’s Recognition Award (AMA PRA) credits are accepted in the US as evidence of participation in CME activities. For further information on this award, please go to https://www.ama-assn.org. The AMA has determined that physicians not licensed in the US who participate in this CME activity are eligible for *AMA PRA* *Category 1 Credits™*. Through agreements that the AMA has made with agencies in some countries, AMA PRA credit may be acceptable as evidence of participation in CME activities. If you are not licensed in the US, please complete the questions online, print the AMA PRA CME credit certificate, and present it to your national medical association for review.

## Article Title:  Risk for *Clostridiodes difficile* Infection among Older Adults with Cancer

## CME Questions

1. **You are advising a large hospital regarding *Clostridiodes difficile* infection (CDI) prevention among older adults. According to the retrospective cohort study using population-based Surveillance, Epidemiology, and End Result Medicare data for 2011 by Kamboj and colleagues, which of the following statements about findings of cohort analysis regarding risk for CDI in older adults is correct? **
  - Among the 93,566 Medicare beneficiaries in the cohort, 3.7% overall had CDI during the study period
  - The proportion of CDI was 2.8% among patients with cancer and 2.4% in patients without cancer in unadjusted analyses
  - Men had a higher proportion of CDI than did women
  - Region of residence did not affect CDI incidence
2. **According to the nested case-control analysis among 2,421 case-patients with CDI and 12,105 control participants matched on age and sex, which of the following statements about factors affecting risk for CDI in older adults is correct?**
  - Risk for CDI was significantly increased for all solid tumors, regardless of time of diagnosis and presence or absence of metastasis
  - Patients with cancer vs patients without cancer did not have significantly higher odds for CDI
  - Regardless of having a cancer diagnosis, ≥2 prior hospitalizations, and a prior skilled nursing facility stay were each associated with increased odds for CDI occurrence
  - Race distribution was significantly different in CDI cases than in controls
3. **According to the retrospective cohort study with a nested case-control analysis by Kamboj and colleagues, which of the following statements about clinical implications of risk for CDI in older adults with cancer is correct? **
  - The study proves that excessive antibiotic use associated with cancer treatment increases risk for CDI
  - This study showed that the burden of CDI in older adults is greater among persons with underlying cancer, which can inform targets for prevention
  - Symptoms and complications of CDI in elderly patients are similar to those in younger patients
  - CDI during cancer treatment does not significantly affect management
